# A Qualitative Study on the Knowledge and Awareness of the Mid-Day Meal Scheme Among Parents and Teachers

**DOI:** 10.7759/cureus.89988

**Published:** 2025-08-13

**Authors:** Rashmi Kulkarni, Priyanka Pareek, Subhadra Mandalika, Shravya Karkera, Chethana Chandrasekar

**Affiliations:** 1 Clinical Nutrition, MGM School of Biomedical Sciences, Mahatma Gandhi Mission Institute of Health Sciences (MGMIHS), Navi Mumbai, IND; 2 Foods, Nutrition and Dietetics, College of Home Science, Nirmala Niketan, Mumbai, IND

**Keywords:** focus groups, india, mid-day meal, nutrition, qualitative research, school feeding

## Abstract

Background

The mid-day meal (MDM) scheme is one of India’s flagship school-based nutrition programs aimed at improving health and education outcomes of children. While numerous quantitative studies have assessed its benefits on the nutritional status of children and their attendance, there is limited exploration of stakeholders’ experiences.

Objective

This study aims to assess the knowledge, perceptions, and experiences of parents and teachers about the MDM scheme in Maharashtra, India.

Methods

Thirty participants, including 20 parents and 10 teachers, were purposively selected from a government-run primary and upper primary school located in Khopoli, Khalapur block, Raigad district, Maharashtra. A qualitative exploratory design was adopted to collect data on the knowledge and awareness of MDM programmes using focus group discussions (FGDs). Data were collected using FGD guides and were analyzed thematically across five domains: awareness, perceived impact, challenges, stakeholder roles, and suggestions for improvement.

Results

Participants viewed the MDM scheme positively in terms of improving classroom attention, reducing hunger, and encouraging school attendance. However, limited awareness of nutritional guidelines, concerns over meal monotony and taste, and the absence of formal feedback mechanisms were recurrent concerns. Teachers reported infrastructural and supervisory challenges, while parents expressed a willingness to contribute more actively.

Conclusion

The MDM scheme is considered beneficial by the parents and teachers, but implementation challenges limit its full impact. Improving meal diversity and institutionalizing community participation can strengthen its effectiveness and sustainability while enhancing stakeholders’ perception about MDM.

## Introduction

Malnutrition and hunger continue to pose significant public health challenges in India, particularly among school-age children. These issues not only affect physical growth but also impede cognitive development and academic achievement [1,2]. To address these interlinked concerns, the Government of India introduced the mid-day meal (MDM) scheme in 1995 under the National Programme of Nutritional Support to Primary Education (NP-NSPE) [3]. The MDM scheme is widely recognized as a key national intervention aimed at reducing classroom hunger and improving nutritional intake among schoolchildren [4]. Research has identified a high prevalence of undernutrition among school-aged children in urban slum settings, underscoring the need for such interventions [5]. At the same time, studies exploring parental perspectives reveal both appreciation for the program's benefits and concerns related to food quality, hygiene, and consistency in implementation [6]. There is a growing body of literature highlighting the wide-ranging benefits of school feeding programs. In Nepal, for instance, the introduction of MDM in public schools has led to better student attendance, higher academic engagement, and a marked reduction in dropout rates over a period of three years [7]. In the Indian context, mandated school meal initiatives have also been shown to improve children's nutritional status and produce long-term gains in cognitive development and school participation [8]. Furthermore, state-level evaluations highlight that well-organized MDM programs can positively influence school enrolment, promote health and hygiene awareness, encourage community participation, and support inclusive learning environments through structured mealtime practices [9]. In the Indian context, MDM programs have been associated with improvements in nutritional outcomes such as body mass index (BMI), particularly among children from disadvantaged socioeconomic backgrounds [10]. Additionally, evidence from India indicates that participation in school feeding programs contributes to enhanced concentration and learning achievement, as well as reduced absenteeism [11].

Although the MDM scheme has been widely assessed using quantitative indicators such as nutritional outcomes, school enrolment, and retention rates, there is a clear gap in the literature concerning qualitative perspectives from key stakeholders. Teachers play a central role in the day-to-day supervision and implementation of the program, and parents observe its effects on children within the home environment [12].

A deeper understanding of these lived experiences is necessary to complement existing quantitative evidence and inform more grounded, stakeholder-sensitive policy decisions. Considering this, the present study explores the knowledge, perceptions, and experiences of parents and teachers about the MDM scheme in a government school setting in Maharashtra, India, had been studied using focus group discussions (FGDs). Specifically, the study aimed to understand how parents and teachers perceive the nutritional, educational, and social outcomes of the MDM scheme; to identify key challenges encountered in its day-to-day implementation; to gather feedback on aspects such as food quality, hygiene, infrastructure, and regularity of meals; and to examine the extent of involvement of all relevant stakeholders in the delivery and monitoring of the program. In doing so, the study sought to generate stakeholder-driven recommendations to improve the design and effectiveness of the scheme at the ground level.

## Materials and methods

Study design and participants

A qualitative exploratory design was adopted to assess the knowledge, perceptions, and experiences of parents and teachers regarding the MDM scheme. The study was conducted in a government-run primary and upper primary school located in Khopoli, Khalapur block, Raigad district, Maharashtra.

Participants (n = 30) with direct experience of the scheme, including parents (n = 20) of children enrolled in Grades 1-7 and teachers (n = 10) who had at least six months of teaching experience and were actively involved in monitoring the implementation of the MDM scheme, were selected using the purposive sampling technique. Informed written consent was obtained from all participants before participation. A total of three FGDs were conducted. FGD 1 with teachers (Teachers Group), FGD 2 with parents (Parents Group A, n = 10), and FGD 3 with a second set of parents (Parents Group B, n = 10). These groupings were designated to facilitate comparative thematic analysis and to capture a wider range of experiences and perspectives.

Data collection

Sociodemographic profile was collected through the structured questionnaire (Appendix 1). Socioeconomic status was assessed using the Kuppuswamy socioeconomic scale, which is an open-access tool and freely available for academic use [13] (Appendix 1, Tables 2-5). National MDM scheme guidelines were utilized to formulate the FGD guides [3]. FGD guides were developed separately for parents and teachers (Appendix 1). Each guide had 14 open-ended questions covering scheme awareness, impact on children’s health and education, operational challenges, stakeholder roles, and suggestions. Discussions, conducted in Marathi during school hours in familiar school settings, lasted 60-90 minutes. The principal investigator facilitated the FGDs with a trained note-taker. Sessions were audio- and video-recorded with consent, and non-verbal cues were captured through field notes.

Data analysis

Transcripts were first prepared in Marathi and then translated into English by bilingual researchers. To ensure the accuracy of the translation, selected portions were back-translated and checked for consistency in meaning.

The data were analyzed using a manual, inductive thematic approach, where patterns and ideas were drawn directly from what participants said. Two researchers read the transcripts carefully and carried out line-by-line coding, identifying important points and recurring topics. After coding independently, they compared their codes, discussed any differences, and agreed on a common set of codes. This process helped ensure inter-coder reliability by confirming that both researchers interpreted the data in a consistent and transparent way.

The codes were then grouped into broader topics, which were developed into main themes. These themes were refined through multiple discussions and by going back to the data to ensure they accurately reflected what participants shared. Common themes included meal monotony, food quality, infrastructure issues, and the lack of feedback channels. Notes taken during the FGDs were also used to understand the context better and support the findings.

## Results

Sociodemographic profile

*Teachers* 

The study included 10 teachers, all were actively involved in MDM supervision. The majority (9, 90%) were females, while one (10%) was a male. The mean age of the teachers was 42 ± 4.8 years. Most were graduates or postgraduates, with formal training in education or related fields and belonged to the upper socioeconomic group.

Parents

A total of 20 parents participated in the study, comprising four (20%) males and 16 (80%) females. All parents belonged to the lower socioeconomic group. The mean age of the parents was 36 ± 5.2 years. In terms of education, most parents had studied up to secondary or higher secondary level. Among them, 12 (60%) were engaged in heavy manual work such as construction or factory jobs, while the remaining eight (40%) worked as unskilled labourers, including domestic helpers and daily wage workers.

Focus group discussion

Data from three FGDs (with 20 parents and 10 teachers) were analyzed thematically. The analysis revealed five core themes related to awareness, impact, challenges, stakeholder involvement, and suggestions for improvement. A summary of the themes and sub-themes is presented in Table 1.

**Table 1:** Themes and sub-themes identified through thematic analysis

Main Themes	Subthemes (Teachers)	Subthemes (Parents)
Awareness of the MDM Scheme	General awareness	General awareness
	Nutritional guidelines	Nutritional objectives
	Awareness of fortified rice	Awareness of fortified rice
Perceived Impact of the MDM Scheme	School attendance	Academic improvement
	Classroom behaviour	Household relief
	Energy and participation	Energy and study habits
	Structured routine	Social participation
	Group engagement	School Motivation
Concerns and Challenges of the MDM Scheme	Meal monotony	Limited menu awareness
	Portion inadequacy	Meal monotony
	Poor hygiene	Portion inadequacy
	Delayed food supply	Hygiene and sanitation
	Infrastructure gaps	
Stakeholder Roles and Involvement in the MDM Scheme	Meal supervision	Limited formal feedback
	Lack of training	Desire for structured meetings
	Minimal parent interaction	
	No formal reporting	
Suggestions for Improvement	Infrastructure improvements	Meal variety
	Staff training	Seasonal foods
	Local audits and monitoring	Local sourcing
		School kitchen gardens
		Parental participation

FGD-Theme 1: Awareness of the MDM Scheme

The following are the awareness of the MDM scheme of teachers in FGD Group 1.

General awareness of the MDM scheme: All teachers (10, 100%) acknowledged that the MDM scheme is a government initiative aimed at providing hot meals to students during school hours. However, only two (20%) teachers recognized that the scheme’s objective includes addressing children’s daily nutritional needs. The majority (8, 80%) perceived the scheme mainly as a strategy to increase enrolment and regular attendance, particularly among students from economically disadvantaged backgrounds.

Awareness of fortified rice: None of the teachers were aware of the inclusion of fortified rice in the school meals. When this information was shared during the FGD, all participants expressed concern over the lack of formal communication regarding the use, benefits, or preparation methods of fortified rice.

Knowledge of nutrition standards: Teachers did not know the recommended nutrition standards or age-specific portion sizes. This indicated a significant information gap concerning the dietary framework of the scheme.

Communication and awareness initiatives: All teachers reported that the school had never conducted awareness sessions or distributed written information about the scheme’s nutritional goals or its specific components. This lack of engagement left them uninformed about the essential aspects of the MDM scheme beyond its logistical execution.

The following are the awareness of the MDM scheme of parents in FGD Groups A and B.

General awareness of the MDM scheme: Both groups of parents were aware of the free meal distribution at school by the government. In both groups, almost 14-16 (70-80%) of the parents learnt about the scheme through their children, while the rest were informed by neighbors or community members.

Understanding of nutritional objectives: Despite being aware of the scheme’s existence, knowledge about its nutritional purpose was limited. Only two (10%) parents in each group acknowledged that the scheme aimed to address children’s dietary needs, highlighting a widespread lack of understanding regarding its health-related goals.

Awareness of fortified rice: None of the parents in either group had any prior knowledge about fortified rice being used in school meals. When the concept was explained during the discussion, all participants were surprised. Concerns were raised about the safety of adding micronutrient-fortified rice to the food without parental knowledge, with some equating it to adulteration. Parents also reported that they had never been informed about the presence or benefits of such ingredients.

Knowledge of meal preparation and food safety: Parents did not have knowledge about meal preparation or any food safety and hygiene standards followed in the school kitchen, which led the apprehensions about the MDM.

Communication and transparency from schools: No verbal or written communication had been shared by the school authority regarding the scheme’s objectives, meal content, or preparation processes. Group B parents specifically pointed out that menus, nutritional information, and food safety updates had never been provided. Several parents expressed concern about the overall lack of transparency, with one parent remarking on their complete reliance on their children's feedback about the meals.

FGD-Theme 2: Perceived Impacts of the MDM Scheme

The following are the perceived impacts of the MDM scheme on teachers in FGD Group 1.

Effect on school attendance: All teachers (10, 100%) reported that the MDM scheme had a positive effect on children’s school attendance.

Classroom behavior: In terms of classroom behaviour, nine (90%) teachers reported an improvement in students' concentration and focus after eating. They observed that children were more attentive during post-lunch classes.

Energy levels and classroom participation: All teachers (10, 100%) shared that children appeared more energetic and alert after eating, and this positively affected their participation in classroom discussions and activities.

Daily routine and school day structure: About six (60%) teachers highlighted that the meal had become a motivating aspect to follow the daily routine of school.

Group learning engagement: Seventy percent (seven) of teachers stated that students were more willing to take part in group learning and cooperative tasks following lunch, indicating an overall increase in engagement with school routines.

The following are the perceived impacts of the MDM scheme on parents in FGD Groups A and B.

Focus and academic improvement: Among parents in Group A, eight (80%) observed that their children appeared more focused and active in the afternoon after consuming the school meal. They also reported that their children showed improved attention to classwork, greater interest in completing homework, and increased engagement when sharing about their school experiences at home. About six (60%) parents from Group B stated that the meal routine contributed to structuring their daily schedule. One parent explained, “Now they wake up on time, get ready without complaining, and come home without fatigue.”

Reduction in household burden: Parents in Group B reported that the scheme reduced the burden of meal preparation at home. All parents (10, 100%) in this group mentioned that having lunch provided at school allowed them to save both time and money, which could be redirected toward other household priorities.

Energy and evening study habits: Seventy percent (14) of the parents from both groups observed that their children were more energetic after school, which positively influenced evening study habits.

Social and classroom participation: Furthermore, 10 (50%) from both groups mentioned that their children were more socially involved, showed increased participation in classroom activities, and returned home with enthusiasm for school.

Motivation and attendance: In both groups, all parents (20, 100%) agreed that the MDM scheme positively impacted school attendance. Group A parents reported that their children became more enthusiastic about attending school. Meanwhile, parents in Group B viewed the scheme as a strong motivator for regular attendance, particularly among children from food-insecure households. A few parents also mentioned that it may have contributed to reducing dropout rates by ensuring at least one dependable meal per day.

FGD-Theme 3: Concerns and Challenges of the MDM Scheme

The following are the concerns and challenges of the MDM scheme for teachers in FGD Group 1.

Monotony: All teachers (10, 100%) reported that the prescribed weekly menu, which included dal-rice, pulao, legumes, sprouts, and khichdi, was followed. However, 90% observed that meals were highly repetitive, leading to frequent complaints from students and reduced interest in consumption.

Lack of portion size specifications: Concerns about portion adequacy were raised by all teachers (10, 100%). Meals were served uniformly without consideration for age or dietary needs, as none of the teachers had received training on recommended portion sizes.

Poor hygiene and paucity of vessels: Seventy percent (seven) of teachers expressed concerns, including the absence of handwashing facilities, poor utensil cleaning, and unsafe serving practices. About three (30%) teachers reported that helpers occasionally served two students on the same plate to minimize utensil use - an approach seen as unhygienic and inappropriate.

Delayed food supply: Delays in food supply were reported by eight (80%) teachers, often due to late delivery or staff shortages. These interruptions led to postponed meals, disrupting class schedules and affecting students’ post-lunch engagement.

Inadequate infrastructure and staffing: All teachers (10, 100%) identified infrastructure and staffing limitations as critical barriers. Schools often lacked proper kitchens, storage, clean water, and trained support staff. Teachers were expected to supervise meals without formal guidance, adding to their academic workload.

The following are the concerns and challenges of the MDM scheme for parents in FGD Groups A and B.

Limited awareness of weekly menu: Parental awareness of the prescribed weekly menu remained limited. In Group A, only two (20%) of parents reported being aware of the scheduled meals, while, in Group B, awareness was even lower (1, 10%). In both groups, parents noted that they had not received any formal communication from the school regarding the meal plan.

Meal monotony: All parents from both groups (20, 100%) reported that their children frequently complained about the repetitiveness of meals, particularly the daily repetition of rice and dal. Many parents expressed concern that this lack of variety reduced children's interest in the meals and, in some cases, led to refusal or skipping of food.

Concerns about portion size adequacy: Perceptions of inadequate portion size were common among both groups. In Group A, nine (90%) parents believed that the amount of food served was insufficient, particularly for older children. Similarly, all parents (10, 100%) in Group B reported concerns that their children asked for more food shortly after school.

Hygiene and sanitation issues: Hygiene-related concerns were also prominent. In Group A, six (60%) parents reported complaints from their children regarding unclean utensils, lack of handwashing facilities, and improper food handling. Group B parents reported similar concerns, with seven (70%) mentioning poor sanitation practices during meal distribution.

Theme 4: Stakeholder Roles and Involvement in the MDM Scheme

The following are the stakeholder roles and involvement of the MDM scheme for teachers in FGD Group 1.

Operational involvement in meal distribution: All teachers (10, 100%) reported being responsible for daily supervision of meal distribution. Their tasks included ensuring timely service, managing student queues, and monitoring neatness during lunch. However, their involvement was operational rather than strategic, and none of the teachers were involved in planning menus, managing supplies, or assessing food quality.

Absence of training and guidelines for meal supervision: None of the teachers received any training specifically on portion standards, nutritional value, or hygiene protocols. As a result, meal supervision was carried out informally and without referring to standardized guidelines.

Limited parent engagement and lack of formal reporting: Only one (10%) teacher had interacted with parents about the scheme, and those interactions were situationally planned and informal. There was no system in place for structured reporting or review of meal-related observations.

The following are the stakeholder roles and involvement of the MDM scheme for parents in FGD Groups A and B.

Several parents (12, 60%) from both groups suggested that a simple monthly meeting or feedback session would allow them to share concerns and offer suggestions.

Theme 5: Suggestions for Improvement

The following are the suggestions for improvement of teachers in FGD Group 1.

Improvement in school infrastructure: All teachers (10, 100%) recommended infrastructural improvements, including a dedicated, enclosed kitchen, proper ventilation, storage space for grains, and access to clean water. These were seen as essential to ensure hygiene and streamline meal preparation.

Training for staff on meal management: Eighty percent (8) of teachers emphasized the need for regular training for both teaching and kitchen staff. Suggested areas included food hygiene, nutrition basics, portion size guidelines, and supervisory protocols. Teachers believed that such training would strengthen their role in maintaining meal quality.

Involving local stakeholders: Sixty percent (six) suggested involving local stakeholders, such as school management committees or community volunteers, to periodically monitor meal distribution. Teachers also proposed introducing third-party audits to review hygiene and quality standards.

The following are the suggestions for improvement of parents in FGD Groups A and B.

Enhancing meal variety through seasonal foods: In Group A, seven (70%) parents recommended incorporating seasonal vegetables and fruits into the existing menu to enhance both the nutritional value and appeal of the meals. They believed that a more diverse and colourful meal would not only improve children's health but also increase their interest in consuming the food regularly, thereby reducing food wastage. Similarly, in Group B, all parents (10, 100%) advocated for the regular inclusion of seasonal fruits and green vegetables to improve the taste and nutritional content of meals. Moreover, eight (80%) Group B parents emphasized that meals would become more acceptable if food items were diversified according to seasonal availability, as well as children’s preferences and local dietary habits.

Promoting freshness through local sourcing: Regarding the sourcing of ingredients, four (40%) Group A parents proposed that food should be procured from local markets or nearby farmers. They believed that this approach would ensure fresher meals, reduce supply delays, and contribute to the local economy. Additionally, three (30%) of Group A parents suggested the establishment of school kitchen gardens. This initiative was seen as a practical, hands-on method to not only improve the freshness of ingredients but also promote nutrition education and sustainable food practices among students.

Parental participation in the MDM scheme: Parental engagement was particularly strong in Group B, where all parents (10, 100%) expressed a willingness to participate in the program through voluntary roles. These included attending feedback meetings and offering suggestions. Parents emphasized that such active involvement would significantly enhance the program’s transparency, responsiveness, and overall effectiveness.

## Discussion

This qualitative study explored the knowledge and perceptions of parents and teachers regarding the MDM scheme in a government school. The findings highlight both program strengths and areas needing improvement, offering insight into how local stakeholders experience its implementation.

In this study, a structured qualitative approach guided the thematic analysis. Inductive coding and constant comparison techniques were used to develop meaningful categories and identify patterns across participant responses. Thematic findings revealed that the MDM scheme was widely regarded as a driver of improved school attendance, greater attentiveness in class, and enhanced academic performance, particularly for children from low-income households - views that align with previous research on the scheme’s educational impact [14]. Broader evaluations have also linked the program to increased enrolment and improved retention rates among socioeconomically disadvantaged children [15]. While most participants understood the scheme as a government-sponsored food initiative, there was limited awareness of its intended nutritional or health objectives. This lack of clarity reflects persistent gaps in stakeholder understanding observed in other studies [16].

Participants repeatedly raised the issue of limited opportunities for formal engagement. Parents expressed a desire to contribute suggestions, but no structured platform was in place for doing so. Teachers, too, indicated that they had received no refresher training and lacked clear guidance on how to involve parents or monitor meals. These findings are consistent with prior qualitative studies that document limited parent involvement, lack of structured consultation, and insufficient institutional support as key barriers to effective community engagement in the MDM scheme [17].

Similar findings have been observed in a qualitative case study from a rural school in Odisha, where teachers, parents, and school management committee members noted that the MDM scheme had improved enrolment and attendance, but suffered from irregular food quality, low protein content, and poor infrastructure for serving meals. Despite strong community support, the responsibility placed on teachers for meal oversight and the irregular release of funds contributed to dissatisfaction among both parents and staff [18].

Teachers, despite being responsible for meal supervision, had not received formal instruction on portion sizes, nutritional standards, or hygiene protocols - highlighting a disconnect between administrative expectations and ground-level realities [19,20]. Similarly, parents had little access to accurate information about the scheme. Their understanding was often based on what children shared at home or what they observed themselves, rather than through any official communication. As a result, many parents were unsure about the scheme’s impact on their child’s nutrition or academic progress.

Despite these challenges, participants acknowledged the value of the program in the rural school context. They emphasized its role in addressing hunger, supporting school attendance, and improving focus in the classroom - particularly among children from underprivileged backgrounds [21].

To ensure trustworthiness, the analysis was guided by core qualitative criteria: credibility, transferability, dependability, and confirmability. This was supported by prolonged engagement with the data, peer debriefing, and a maintained audit trail to enhance transparency and rigor.

Another concern shared by many participants was the lack of variety in meals. Parents noted that children were often served the same food repeatedly - mostly rice, dal, and simple vegetables - which sometimes led them to skip meals. For some families, however, this remained the only substantial meal the child would receive in a day. Concerns about taste, freshness, and overall appeal of the meals were also frequently raised, with parents and teachers stating that children occasionally left food unfinished due to dissatisfaction.

Teachers highlighted various logistical challenges in day-to-day implementation. These included delayed delivery of ingredients, lack of permanent cooking staff, and poor kitchen facilities. As a result, teachers often had to divide their time between classroom responsibilities and managing the meal process, which negatively impacted both teaching and program oversight [12].

In addition to operational concerns, many participants pointed to the absence of regular feedback mechanisms at the school level. Although parents and teachers were open to improving the program, there was no structured way to communicate their concerns or suggestions. Teachers also noted the lack of support from the administration in terms of refresher training or updated guidance, which limited their ability to adapt or improve practices. This is contrary to the scheme’s stated aim of fostering community participation and accountability [16].

Participants recommended expanding the menu to include more diverse and protein-rich items, such as lentils, eggs, or seasonal vegetables. Several parents and teachers also suggested linking the program with school gardens and basic nutrition education, to encourage healthy eating habits and increase children’s understanding of food sources [3].

These findings align with broader policy evaluations of the MDM scheme, which have consistently highlighted structural limitations - including inadequate hygiene monitoring mechanisms, infrastructural deficits, and weak nutritional surveillance systems. If these structural issues are not addressed through more inclusive and locally grounded decision-making, the scheme will continue to underdeliver, regardless of how widely it is implemented [22]. Finally, the importance of local ownership and school-level monitoring was emphasized. Many participants believed that involving community members more actively - through feedback collection, school garden activities, or menu planning - would improve both implementation and student engagement. These suggestions reflect a broader recognition that locally responsive solutions are essential to fulfilling the scheme’s goals in rural, resource-limited settings.

Strengths

The following are the strengths of the study: It (1) included both parents and teachers to get a well-rounded view of how the MDM scheme works and is perceived; (2) used FGDs to gather meaningful information that might not come out in surveys; (3) followed a step-by-step coding method; and (4) highlighted practical challenges and local suggestions that match national policy goals such as Pradhan Mantri Poshan Shakti Nirman (PM POSHAN), giving useful input for policymakers and school leaders.

Limitations

The following are the limitations of the study: (1) in FGDs, dominant voices may have influenced the discussion, potentially limiting input from quieter participants; and (2) the study was conducted in a single government school setting, which may limit the generalizability of findings to other regions or school contexts with different administrative or community dynamics.

## Conclusions

The MDM scheme is widely recognized for its positive effects on child health, nutrition, and school attendance. This study identified a significant gap in stakeholder awareness. Many parents and teachers were unaware of the scheme’s dietary goals and its role in supporting children's physical and cognitive development. This points to a need for better communication and structured stakeholder involvement to bring ground-level practices in line with policy interventions. The limited variety of food has affected children’s interest in eating. Therefore, broader dietary planning that incorporates local preferences, seasonal foods, and culturally acceptable food sources - alongside infrastructure and supply chain improvements - is imperative.

Beyond regular meal delivery, the long-term impact of the scheme depends on how meaningfully schools, teachers, and parents are involved in its day-to-day functioning. Bridging the gap between centralized planning and local realities requires stronger infrastructure, regular training, and active feedback mechanisms that allow communities to participate in shaping how the scheme works for them. These findings reinforce the importance of listening to those closest to implementation. Including the voices of parents and teachers is not just important for smoother execution - it is essential for making the scheme more responsive, inclusive, and sustainable. At the end, the success of the MDM scheme depends not only on what is served, but on how well it is understood, supported, and shaped by the communities it is meant to serve.

## Appendices

FGD guides for parents and teachers

Name of Region:

District:

Village:

Number of Focus Group Participants:

Date of FGD:

Name of Interviewer:

Name of Moderator:

Name of Recorder:

Name of Supervisor / Institution:

Date of Data Transcription:

Name of Transcriber:

Signature of Supervisor/Institution:

Date:

Time:

Place:

General Information of the Participants (Parents)

Name of Parent:

Age:

Gender:

Name of Child:

Age:

General Information of the Participants (Teachers)

Name of Teacher:

Age:

Gender Class:

**Table 2:** Modified Kuppuswamy Socioeconomic Status Scale (2024) The Kuppuswamy socioeconomic scale is an open-access tool and freely available for academic use [13].

S. No.	Education of the Head	Score
1	Profession or honours	7
2	Graduate	6
3	Intermediate or diploma	5
4	High school certificate	4
5	Middle school certificate	3
6	Primary school certificate	2
7	Illiterate	1

**Table 3:** Occupation of the Head

S. No.	Occupation of the Head	Score
1	Legislators, senior officials and managers	10
2	Professionals	9
3	Technicians and associate professionals	8
4	Clerks	7
5	Skilled workers and shop/market sales workers	6
6	Skilled agricultural and fishery workers	5
7	Craft and related trade workers	4
8	Plant and machine operators and assemblers	3
9	Elementary occupation	2
10	Unemployed	1

**Table 4:** Family Monthly Income

S. No.	Income Range (2012)	Income Range (2024)	Income Range (2016)	Income Range (2024)	Score
1	≥ 30,375	≥ 1,25,753	≥ 51,646	≥ 2,13,814	12
2	15,188-30,374	62,875-1,25,752	25,811-51,645	1,06,850-2,13,813	10
3	11,362-15,187	47,038-62,874	19,351-25,809	80,110-1,06,849	6

**Table 5:** Socioeconomic Class

S. No.	Socioeconomic Class	Score Range
1	Upper (I)	26–29
2	Upper Middle (II)	16–25
3	Lower Middle (III)	11–15
4	Upper Lower (IV)	5–10
5	Lower (V)	<5

Appendix 1: Focus Group Discussion Guide for Parents

1. What do you know about the mid-day meal program offered at your child's school?

2. Are you aware of the nutritional content and variety of food provided in the mid-day meals?

3. How did you become aware of the existence of the mid-day meal program in the school?

4. What are your thoughts and feelings about the mid-day meal program's impact on your child's health and academic performance?

5. Do you believe the program is beneficial in promoting regular attendance and reducing drop-out rates among students?

6. How important do you consider the mid-day meal program for your child's overall well-being?

7. How often do you discuss the quality and content of the mid-day meals with your child?

8. Do you observe any changes in your child's eating habits or behavior after consuming the mid-day meals?

9. Are there any concerns or suggestions you have regarding the portion sizes or food preferences of the meals?

10. What challenges, if any, do you face in ensuring your child receives the mid-day meals regularly?

11. Are there any factors that positively influence your child's enthusiasm towards the mid-day meal program?

12. How can you contribute to improving the program's implementation and effectiveness?

13. Based on your experience, what improvements would you like to see in the mid-day meal program to better meet your child's nutritional needs?

14. Are there any specific dietary preferences or cultural considerations that could be considered to enhance the meals' acceptance among children?

Appendix 2: Focus Group Discussion Guide for Teachers

1. How familiar are you with the objectives and guidelines of the mid-day meal program?

2. Are you aware of the specific nutritional requirements addressed by the mid-day meals for school children?

3. Have you received any training or guidance on implementing and monitoring the program at your school?

4. What is your perception of the impact of mid-day meals on students' health, concentration, and classroom participation?

5. Do you believe the program has had any influence on students' overall academic performance and attendance?

6. How important do you consider the mid-day meal program as part of the school environment?

7. How involved are you in overseeing the mid-day meal arrangements and ensuring they meet the required standards?

8. Have you noticed any behavioral or academic changes in students after consuming the mid-day meals?

9. Do you observe any challenges in managing the mid-day meal distribution or ensuring equal access for all students?

10. What are the main challenges faced by the school in implementing the mid-day meal program effectively?

11. Are there any factors that have facilitated the successful implementation and acceptance of the program among students?

12. How can teachers contribute to creating a positive and supportive environment for the mid-day meal program?

13. Based on your experience, what improvements or adjustments would you propose to enhance the mid-day meal program's effectiveness at your school?

14. Are there any specific educational components or activities that could complement the nutritional benefits of the mid-day meals?

## References

[1] (2025). Government of India. National family health survey (NFHS-5). https://dhsprogram.com/pubs/pdf/FR375/FR375.pdf.

[2] Black RE, Allen LH, Bhutta ZA (2008). Maternal and child undernutrition: global and regional exposures and health consequences. Lancet.

[3] Bhavan S (2022). Mid-Day Meal Policy. https://pmposhan.education.gov.in/Files/Guidelines/2023/Guidelines%20on%20PM%20POSHAN%20SCHEME.pdf.

[4] Mehta B, Grover K, Kaur R (2013). Nutritional contribution of mid day meal to dietary intake of school children in Ludhiana district of Punjab. J Nutr Food Sci.

[5] Subhaprada C (2015). Nutritional status of government primary school children in an urban slum, Kurnool, Andhra Pradesh. IJCMAAS.

[6] Dey P, Singh Y (2023). Analysis of parental perception towards mid-day meal scheme in government schools of Coochbehar district, West Bengal. SJMARS.

[7] Sapkota K, Dhungel B, Bhusal K (2025). Impact of free mid-day meal program on academic performance in public schools of Nepal. IJIRSS.

[8] Prakasam S (2017). Perceptions of parents and children on implementation of mid-day meal scheme (case study of UT of Chandigarh). J Indian Educ.

[9] Upadhyay B, Geeta K (2019). Opinion and nutritional status of children consuming mid day meal (MDM) in schools in Delhi. Int J Interdiscip Res Innov.

[10] Boriwal S, Mittal K (2025). Perception of beneficiaries of mid-day meal programme and its impact on general health of girl students. Int J Home Sci.

[11] Rani R, Sharma D (2017). Problems faced by teachers in implementation of mid day meal scheme at primary school level in Jammu Province. Int J Dev Res.

[12] Kaushal S (2009). A Study of Best Practices in the Implementation of Mid-Day-Meal Programme in Rajasthan. https://pmposhan.education.gov.in/Files/Initiatives%20&%20Case%20Studies/Mid-day-meal-best-practices-by-savita-kaushal-nuepa.pdf.

[13] Mandal I, Hossain SR (2024). Update of modified Kuppuswamy scale for the year 2024. Int J Community Med Public Health.

[14] Paltasingh T, Bhue P (2022). Efficacy of mid-day meal scheme in India: challenges and policy concerns. Indian J Pub Admin.

[15] Gharge S, Vlachopoulos D, Skinner AM, Williams CA, Iniesta RR, Unisa S (2024). The effect of the mid-day meal programme on the longitudinal physical growth from childhood to adolescence in India. PLOS Glob Public Health.

[16] Raveenthiranathan L, Ramanarayanan V, Thankappan KR (2024). Impact of free school lunch program on nutritional status and academic outcomes among school children in India: a systematic review. BMJ Open.

[17] Singh A, Biswas DS (2021). The midday meal scheme in primary education: achievements and future prospects. Am J Soc Humanit Res.

[18] Mohanty SP (2014). Practice of mid day meal scheme at elementary education level: a case study of a rural elementary school. Scholars J Arts Humanit Soc Sci.

[19] Barnali M (2017). Strengthening right to food for children through MDMS. Int J Adv Soc Sci.

[20] Chutani AM (2012). School lunch program in India: background, objectives and components. Asia Pac J Clin Nutr.

[21] Hasija S. (2025). A study on mid-day meal scheme. A STUDY ON MID-DAY MEAL SCHEME.

[22] Joshi HP, Bohara K (2025). Stakeholder perceptions among school nutrition and mid-day meal programme. Sch J Arts Humanit Soc Sci.

